# Perception of Dental Students of COMS-TH regarding Future of Dentistry in Nepal amid COVID-19 Pandemic

**DOI:** 10.31729/jnma.5115

**Published:** 2020-09-30

**Authors:** Deepanshu Garg, Deepika Kapoor

**Affiliations:** 1 Department of Oral Medicine and Radiology, College of Medical Sciences and Teaching Hospital, Bharatpur, Nepal; 2 Department of Pedodontics, College of Medical Sciences and Teaching Hospital, Bharatpur, Nepal

**Keywords:** *COVID-19*, *dentistry*, *future*, *students*

## Abstract

**Introduction:**

There is a global crisis which has been led by COVID-19. The patients undergoing dental procedures and dental professionals are at higher risk of contracting this disease owing to aerosols generated and a lot of face to face contact during the procedures. The aim of this study was to know the perceptions of dental students of COMS-TH regarding future of dentistry in Nepal amid COVID-19 pandemic

**Methods:**

The present cross-sectional descriptive study was conducted at COMS-TH, Bharatpur by sending an online e-survey questionnaire to 146 dental students out of which 99 responded. The e-survey questionnaire consisted of three parts which consisted of questions about demographics, knowledge about COVID-19 and their perceptions about future of dentistry.

**Results:**

The results of the study depicted that most of the students thought dentistry is good and noble profession and will recommend it to young medical aspirants. Most of them wanted to pursue post graduation courses in future giving preference to Oral and Maxillofacial Surgery.

**Conclusions:**

The study concluded that most of the dental students were satisfied with dentistry as their profession and wanted National Dental Association to fix the minimum charges of each dental procedure. Also there is a need to start more post graduation courses in existing institutions providing dental education as most of the students want to pursue it in future.

## INTRODUCTION

There is a lot of global attention being given to the Human Coronaviruses nowadays after the COVID-19 disease which resulted from novel coronavirus.^1^ Severe Acute Respiratory Syndrome coronavirus 2 (SARS-CoV-2) is the cause of COVID-19 which is a highly contagious viral infection. Fomite transmission and respiratory droplets inhalation are the possible indirect and direct possible transmission routes respectively.^2^

This study was undertaken because the patients undergoing dental procedures and dental professionals are at higher risk of contracting this disease owing to aerosols generated and a lot of face to face contact during the procedures.^1^ American Dental Association has asked the dentists all around the globe to only provide emergency treatment and that too taking additional preventive measures and wearing proper protective gear which has not only made dental procedures difficult to perform but also costlier.

The aim of this study was to know the perceptions of dental students of COMS-TH (College of Medical Sciences and Teaching Hospital) regarding future of dentistry in Nepal amid COVID-19 pandemic.

## METHODS

The present cross-sectional descriptive study was conducted at COMS-TH, Bharatpur by sending an online e-survey questionnaire to all the 146 students currently pursuing BDS course so as to know their perceptions about future of dentistry in Nepal during the COVID-19 pandemic (convenient sampling). The study was initiated in starting of June 2020 with approval from COMSTH-IRC and was completed in mid of month with the preparation of manuscript. The inclusion criteria consisted of the dental students currently studying in COMS-TH. The exclusion criteria consisted of students who were not pursuing BDS course and those who were not willing to participate in the study.

The e-survey questionnaire consisted of three parts. The first section consisted of three questions about demography asking the students their gender, hometown and professional year. The second part consisted of five questions regarding the knowledge of participants about COVID-19 disease. The last section consisted of 10 questions regarding the perceptions of participants about the future of dentistry in Nepal during the COVID-19 pandemic. The written consent was not taken as the participants replying back with the filled questionnaire was implied as consent. The results were tabulated in Microsoft Excel and by using SPSS v 23 software, statistical significance was observed.

## RESULTS

An electronic link toan e-survey questionnaire was forwarded toa total of 146 dental students of COMS-TH out of which 99 respondents filled the questionnaire. And, 79 females and 20 males participated and it was found out that 92 (92.9%) of the participants were following daily news and cases of COVID-19 and were also aware of the current CDC or WHO guidelines regarding the infection control. However, the participants who were aware of CDC guidelines regarding infection prevention and control in dental setting were 63 (63.6%).

When asked whether practicing dentistry is going to be expensive in future, 43 (43.4%) participants voted yes and 2 (2%) voted no whereas 54 (54.5%) participants were not sure. Most of them i.e. 82 (82.8%) participants demanded for fixation of the minimum charges of every dental procedure by National Dental Association. Only 35 (35.3%) of the participants agreed to the increase of dental procedure charges in future whereas 38 (38.3%) were not sure about this decision. Performing the dental procedures wearing proper PPE (face shields, N-95 masks, goggles) in future keeping in mind the COVID-19 pandemic is going to be difficult was acknowledged by 47 (47.4%) participants whereas 32 (32.3%) were not of the same opinion. Seventy-two (72.7%) of participants felt that the number of patients in dental clinics and hospitals is going to increase in future. More than 80% of the participants felt that dentistry is a good and noble profession and they will still recommend the young medical aspirants to take up dentistry as their profession in future. Out of the total participants, 92 (92.9%) wanted to pursue post graduation courses in future whereas 5 (5%) were not sure about their decision (Table 1). Out of those who wanted to pursue post graduate courses, 48 (48.4%)of the participants wanted to opt for Oral and Maxillofacial surgery followed by Orthodontics, Conservative Dentistry, Pedodontics, Oral Medicine and Radiology, Prosthodontics, Community Dentistry, Periodontics and Oral Pathology.

**Table 1:** Perceptions of dental students about future of dentistry after COVID-19 pandemic (n = 99).

Perceptions of dental students about future of dentistry n = 99	Yes	No	Not sure
Practicing dentistry costlier in future	43	2	54
Minimum charges of dental procedures to be fixed	82	1	16
Charges of dental procedures should be increased	35	26	38
Dental procedures difficult to perform wearing PPE	47	32	20
Number of dental patients going to increase	72	8	19
Dentistry is good and noble profession	88	2	9
Recommend dentistry as profession to young medical aspirants	80	8	11
Pursue post graduation courses in future	92	2	5

## DISCUSSION

There is a lot of global attention being given to the Human Coronaviruses nowadays after the COVID-19 disease which resulted from novel coronavirus.^1^ Severe Acute Respiratory Syndrome coronavirus 2 (SARS-CoV-2) is the cause of COVID-19 which is a highly contagious viral infection. Fomite transmission and respiratory droplets inhalation are the possible indirect and direct possible transmission routes respectively.^2^ COVID-19 has an incubation period of 2-14 days and can be transmitted by asymptomatic patients also.^3–5^ It is 65-125 nm in diameter and is made up of single strand of RNA. It enters the into the host cells by binding to the receptors of target cells with the help of outer surface which is composed of crown like spikes. Another factor that helps it to gain entry and target the human host cells is its high affinity to the human angiotensin converting enzyme 2 receptors(ACE 2).^1^

There is a global crisis which has been led by COVID-19.^6–7^ Along with rising health care concerns all around the globe, it has also led to collapse of world's economy. Social stability and global health systems are under immense pressure and this pressure is reflected upon the health workers including those of dental care professionals.^8–10^ As a health care worker enters his professional life, he/she starts facing the challenges. These challenges can be a part of transitional phase from a student life to real professional. Students start feeling this pressure just during their graduation phase. So it becomes very important for a dental undergraduate to know the future professional challenges and take appropriate actions to deal with it as it is almost impossible to change your career once you have entered the professional stream.^11^

It is therefore of utmost importance to know what is going on in the minds of young budding dentists about their future who are yet to come in the field of professional dentistry. While going through the literature, not a single study was encountered which particularly focussed on the thoughts going around in the minds of young dental students about future of dentistry after the pandemic. The current study was carried out to get an idea about the perceptions of dental students about future of dentistry in Nepal after COVID-19 pandemic so that it is helpful for the national dental association to form educational and professional guidelines accordingly.

According to our study, more than 90% of the participants were following daily news and cases of COVID-19 and were also aware of the current CDC or WHO guidelines regarding the infection control. This means that dental students of COMS-TH were well aware of the pandemic caused by COVID-19 all around the globe and they must have been practicing proper hygiene instructions at their homes so as to prevent and stop the transmission of infection. However only 63.5% of the participants were aware about the current CDC guidelines to prevent and control the infection in dental setting. Being budding dentists, they have to complete their graduation and work in dental clinics and hospitals in future. So, authorities should do more efforts to circulate related guidelines in newspapers, magazines, and social media along with sending these guidelines in simple language to the institutions providing dental education.

When asked about whether practicing dentistry was going to be costlier in future because of extra personal protective gear, 43 (43.4%) participants voted yes and 54 (54.5%) were unsure. The above findings suggest that the dental students think that dentistry is going to be costlier may be owing to the use of extra protective gears and extra oral suctions for aerosols. There was almost equal distribution of participants in yes, no and not sure when asked about increasing the charges of dental procedures in future. Most of the participants ie 82 (82.8%) voted that minimum charges of each dental procedure should be fixed by National Dental Association so as to maintain equality all over the nation. The concerned authorities should take necessary steps to fix the minimum charges of each dental procedure so that every dentist of Nepal in future should take adequate precautions of infection control and there is no room for unethical practice to lure the patients by providing treatment at less cost. When asked whether the patients in dental clinics and hospitals are going to increase or decrease in future, 72 (72.7%) participants thought that patients will increase. This might be because of fact that there was a complete nationwide lockdown during COVID-19 outbreak and patients were devoid of proper dental care. So, after this pandemic patients might become aware about their dental health and visit hospitals on a regular basis.

More than 80% of the participants thought that dentistry is still a good and noble profession to take and will recommend young medical aspirants to choose this profession for them. Ninety-two (92.9%) of participants voted that they are going to pursue post graduation courses in future and 48.9% of them wanted to go for Oral and Maxillofacial Surgery followed by Orthodontics, Conservative Dentistry, Pedodontics, Oral Medicine and Radiology, Prosthodontics, Community Dentistry, Periodontics and Oral Pathology. The findings of this study show that most of the dental students wanted to pursue post graduation courses, so more and more institutes providing dental education in Nepal should initiate post graduate courses and particularly the subjects which are more favored by students. Another factor is that most of the countries have restricted immigration and students from Nepal will face difficulty to pursue post graduation abroad. So to save the students from unnecessary hassle, more options of post graduation courses should be available in the country. The study only represents students of COMS-TH which is rather a small sample size, so more similar studies are required to be conducted all over the country to get a generalized view on the situation.

## CONCLUSIONS

The study concluded that most of the dental students were satisfied with dentistry as their profession and wanted National Dental Association to fix the minimum charges of each dental procedure. Also there is a need to start more post graduation courses in existing institutions providing dental education as most of the students want to pursue it in future.

## Conflict of Interest

**None.**

## References

[1] Fallahi H, Keyhan S, Zandian D, Kim S, Cheshmi B (2020). Being a front-line dentist during the Covid-19 pandemic: a literature review. Maxillofac Plast Reconstr Surg.

[2] Lauer S, Grantz K, Bi Q, Jones F, Zheng Q (2020). The Incubation Period of Coronavirus Disease 2019 (COVID-19) From Publicly Reported Confirmed Cases: Estimation and Application. Ann Intern Med.

[3] Backer J, Klinkenberg D, Wallinga J (2020). Incubation period of 2019 novel coronavirus (2019-nCoV) infections among travellers from Wuhan, China, 20-28 January 2020. Euro Surveill.

[4] Khader Y, Al Nsour M, Al-Batayneh O, Saadeh R, Bashier H, Alfaqih M (2020). Dentists' Awareness, Perception, and Attitude Regarding COVID-19 and Infection Control: Cross-Sectional Study Among Jordanian Dentists. JMIR Public Health Surveill.

[5] Bescos R, Casas-Agustench P, Belfield L, Brookes Z, Gabaldón T (2020). Coronavirus Disease 2019 (COVID-19): Emerging and Future Challenges for Dental and Oral Medicine. J Dent Res.

[6] González-Olmo M, Ortega-Martinez A, Delgado-Ramos B, Romero-Maroto M, Carrillo-Diaz M (2020). Perceived vulnerability to Coronavirus infection: impact on dental practice. Brazilian Oral Research.

[7] Ather A, Patel B, Ruparel N, Diogenes A, Hargreaves K (2020). Coronavirus Disease 19 (COVID-19): Implications for Clinical Dental Care. J Endod.

[8] Kamate S, Sharma S, Thakar S, Srivastava D, Sengupta K, Hadi A (2020). Assessing Knowledge, Attitudes and Practices of dental practitioners regarding the COVID-19 pandemic: A multinational study. Dent Med Probl.

[9] Spagnuolo G, De Vito D, Rengo S, Tatullo M (2020). COVID-19 Outbreak: An Overview on Dentistry. Int J Environ Res Public Health.

[10] Modi P, Nair G, Uppe A, Modi J, Tuppekar B, Gharpure A (2020). COVID-19 Awareness Among Healthcare Students and Professionals in Mumbai Metropolitan Region: A Questionnaire-Based Survey. Cureus.

[11] Fita S, Alshuraim F, Almulhim A, AlHumaid J, Alhareky M, Nazir M (2020). Possible Future Career Challenges and Associated Factors among Dental Students and Interns. Int J Dent.

